# Optimising Camera Trap Surveys for the Rocky Landform Specialists of the Mount Isa Inlier, Queensland, Australia

**DOI:** 10.1002/ece3.72170

**Published:** 2025-09-25

**Authors:** Jarrad C. Barnes, Elizabeth A. Brunton, Mark G. Sanders, Christofer J. Clemente

**Affiliations:** ^1^ University of the Sunshine Coast Sippy Downs Queensland Australia; ^2^ EcoSmart Ecology Everton Park Queensland Australia

**Keywords:** camera trap, detection probability, occupancy analysis, saxicoline fauna, survey optimisation

## Abstract

Australia's rocky landforms provide critical habitats for a diverse fauna, yet many regions remain underrepresented in ecological research. The Mount Isa Inlier IBRA bioregion, characterised by extensive rocky landscapes, hosts a community of specialist fauna, yet knowledge gaps hinder conservation and management efforts. Building upon previous research on one rock‐dwelling species from the bioregion, the Carpentarian Pseudantechinus ( *Pseudantechinus mimulus* ), the aim of this study was to determine the optimum camera trap timing and duration for a further 11 species and provide a framework for conducting targeted research on this community. Field surveys were conducted at 60 camera stations across nine sampling periods over a total survey effort of 21,965 camera‐days. We investigated all 11 specialists within single‐species, multi‐season occupancy models to assess species‐specific changes in detection probability in response to sampling period and days since baiting and assessed additional detectability parameters including days since baiting, detection rate, diel activity patterns and lunar illumination. Our results highlight temporal variations in detectability, with the optimum timing and duration of surveys differing between specialists and showcasing varying activity patterns and responses to lunar illumination. While camera traps were effective for most specialists, alternative methods may be required for some species with low detectability. We ultimately summarise our findings into a handy, user‐friendly summary table outlining the optimum time of year, survey duration, camera type, hours of operation, lunar illumination scenario and bait type to provide a comprehensive reference to conducting camera trap surveys targeting the community. This paper reflects a growing need for species‐specific survey methodologies to enhance efficiency in remote, data‐deficient regions such as the Mount Isa Inlier and should be particularly relevant to practitioners seeking to assess population trends and inform long‐term conservation efforts.

## Graphical abstract

This study details an extensive camera trap survey of rocky landform fauna in the Mount Isa Inlier, identifying species‐specific detectability patterns. Our findings inform a comprehensive reference table of optimal camera trap survey guidelines to improve survey efficiency for a poorly represented fauna inhabiting a remote, data‐deficient region.

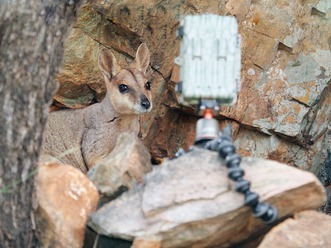

## 1 Introduction

Australia's rocky landforms support a diversity of saxicoline (rock‐dwelling) fauna (Lindenmayer Michael and Lindenmayer 2018). While the most complex and rugged rocky areas emerge as strongholds for a range of endemic and threatened species, particularly mammals and reptiles (e.g., Trainor et al. 2000; Telfer et al. 2008; McDonald et al. 2015; Einoder et al. 2018; Moore et al. 2019), even small, isolated outcrops are important for supporting high levels of diversity, especially in fragmented landscapes (e.g., Michael et al. 2008). Despite their often‐inordinate importance, particularly in disturbed landscapes, rocky landforms across Australia are not equally represented in the scientific literature. Some regions are incredibly well studied, such as the Pilbara (Pepper et al. 2013), the Kimberley (Radford et al. 2014) and the granite inselbergs of Western Australia (Withers and Edward 1997), but others have received little to no attention to date.

The Mount Isa Inlier IBRA bioregion of northwest Queensland comprises an extensive system of rocky landforms that remains largely underrepresented. Rocky landforms account for ~65% of the bioregion and these areas, due to their prevalence and resilience to disturbance, are likely to be of increasingly disproportionate importance to the many fauna species that inhabit the bioregion under pressure from landscape‐scale threats such as overgrazing, weed invasion and changing fire regimes (Crowley 2016). Despite this, most wildlife research in the bioregion has been restricted to a handful of single‐species studies (e.g., Burnett et al. 2014; Harrington et al. 2017) or broader grey literature baseline studies conducted for mining operations (Griffiths 1998; Baker and Griffiths 2005; Perkins et al. 2009). This is largely due to remote access and landholder constraints, with publicly accessible land covering only 2.8% of the bioregion. There are therefore large gaps in current understanding of even the most basic ecological data for many of the taxa of the bioregion, making it difficult to implement meaningful forward‐thinking conservation or management strategies.

We have previously identified a community of specialist fauna inhabiting the rocky landforms of the Mount Isa Inlier bioregion and documented the spatial and temporal habitat use of 12 species (Barnes et al. 2025). We have additionally developed optimised camera trap survey guidelines for one of these, the Carpentarian Pseudantechinus ( *Pseudantechinus mimulus* ; Barnes et al. 2023) and used these guidelines to conduct extensive distributional and habitat surveys (Barnes et al. 2024). Using *P. mimulus* as proof‐of‐concept, this paper applies our framework to develop guidelines for optimised camera trap surveys of the remaining 11 rocky landform specialists. The general scarcity of information about these specialists means efficiently conducting surveys and monitoring programmes, as well as determining potential changes to populations or conservation status in the face of environmental and climate change, is problematic. The aim of this study was therefore to determine when this specialist community is most readily detected on camera traps and to provide an accessible framework to guide targeted research to allow practitioners to optimise survey efficiency in a remote bioregion in which doing so is often time‐ and resource‐intensive.

## 2 Materials and Methods

### 2.1 Study Area and Survey Conditions

The study area lies within the Mount Isa Inlier IBRA bioregion. The bioregion is dominated by rugged rocky hills and ranges between 300 and 500 masl, with vegetation primarily consisting of low open eucalypt woodland and *Acacia* shrubland with a hummock grass (*Triodia* spp.) ground stratum. The climate is characterised by hot, monsoonal summers and dry winters with warm days and cool nights. Mean annual temperatures are 17.3°C–32°C (range = 8.7°C–37.3°C; Bureau of Meteorology 2024). Rainfall is summer‐dominant with an annual mean of 459.8 mm (Bureau of Meteorology 2024). During this study, average daily temperatures fluctuated similarly to long‐term means. Wet season rainfall totals prior to the commencement of the study were below average at ~390 mm (Bureau of Meteorology 2024). Total rainfall during the study exceeded the long‐term mean at ~599 mm (Bureau of Meteorology 2024).

### 2.2 Field Surveys

**FIGURE 1:**
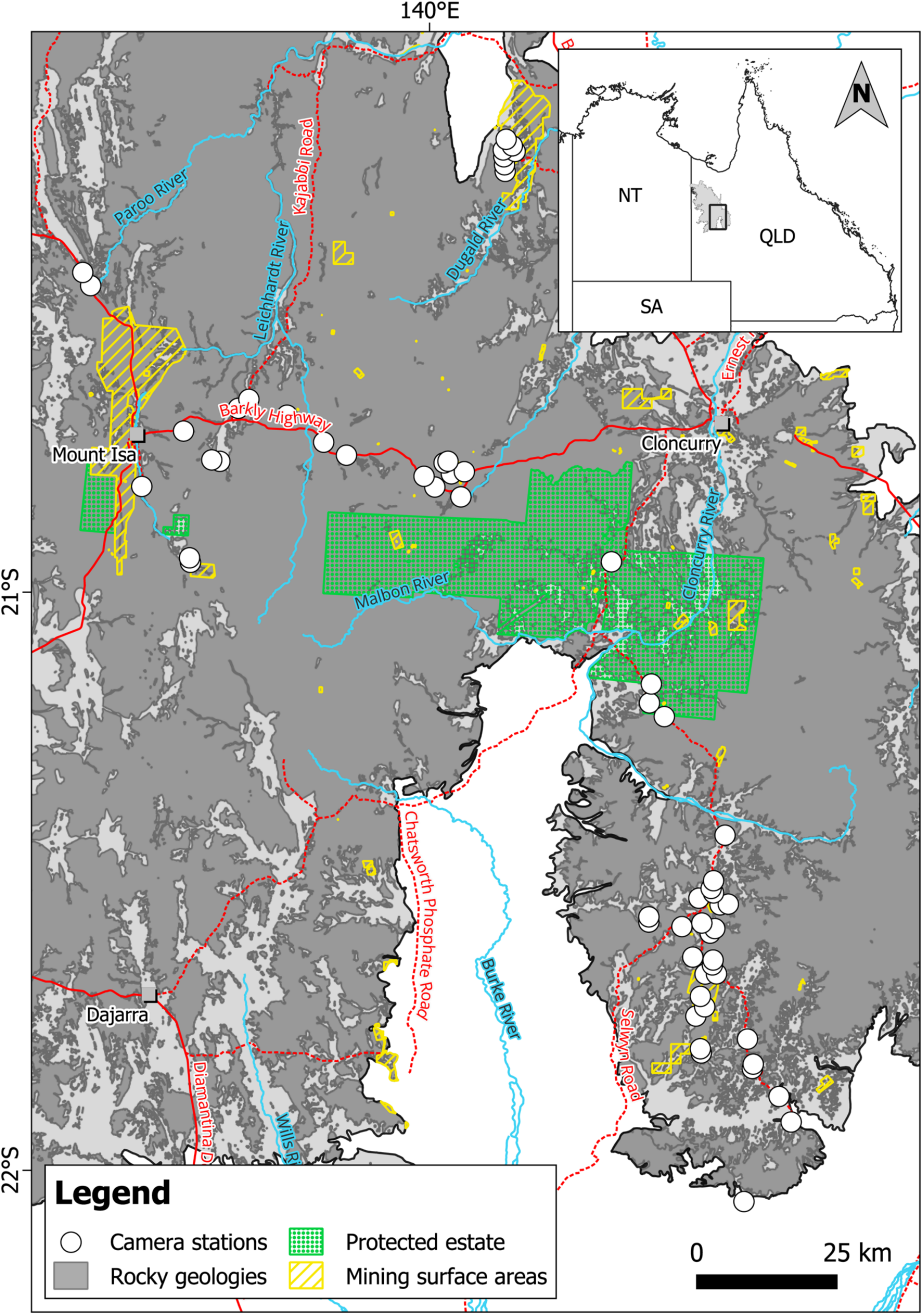
Camera station locations, extent of rocky geologies (Geoscience Australia 2012), major roads, major rivers, population centres, and extent of mining surface leases and protected estate within the study area. With the exception of residential and commercial properties in mapped population centres, the remainder of the study area is pastoral land (primarily cattle grazing). Inset shows the location of the Mount Isa Inlier within the state of QLD.

Field surveys investigated the presence of vertebrate fauna on rocky landforms at 60 camera stations across an area of 11,523 km^2^ (Figure 1). Nine sampling periods were conducted from May 17, 2018, to June 10, 2019 (Table 1), by repeated targeted surveys with a camera service interval of 27–68 days (mean ± SD = 50.88 ± 11.72 days) over a total survey effort of 21,965 camera‐days. All camera stations were located on rocky landforms and consisted of a single ScoutGuard/BolyGuard SG562‐C baited with ~125 g of peanut butter smeared directly onto the rock surface at 90–159 cm (mean ± SD = 115.46 ± 13.56 cm) in front of the camera. Distance to nearest neighbouring camera station ranged from 772 to 24,462 m (mean ± SD = 3265 ± 4026). Cameras were set to 24‐hour operation, ‘High’ sensitivity, three captures per trigger and a 0‐second delay between photos within each trigger. For further details, see (Barnes et al. 2023, 2025).

**TABLE 1:** Survey effort across nine sampling periods with a service interval of 27–68 days.

Sampling period	Survey period	Active cameras	Camera‐days
May 2018	17/05/2018–07/06/2018	60	1240
July 2018	07/07/2018–28/07/2018	60	1256
August 2018	16/08/2018–06/09/2018	60	1210
October 2018	01/10/2018–22/10/2018	59	1227
November 2018	15/11/2018–06/12/2018	59	1219
January 2019	02/01/2019–23/01/2019	60	1244
March 2019	02/03/2019–23/03/2019	56	1135
April 2019	31/03/2019–21/04/2019	58	1208
May 2019	15/05/2019–05/06/2019	59	1227

*Note:* ‘Survey period’ is a date range from deployment/service of the first camera to service/collection of the last camera in each sampling period. ‘Camera‐days’ reflects the total number of days over which the pool of cameras was active after truncating the dataset to 21 days per sampling period and correcting for camera failure/malfunction.

### 2.3 Camera Trap Imagery Processing

**FIGURE A1:**
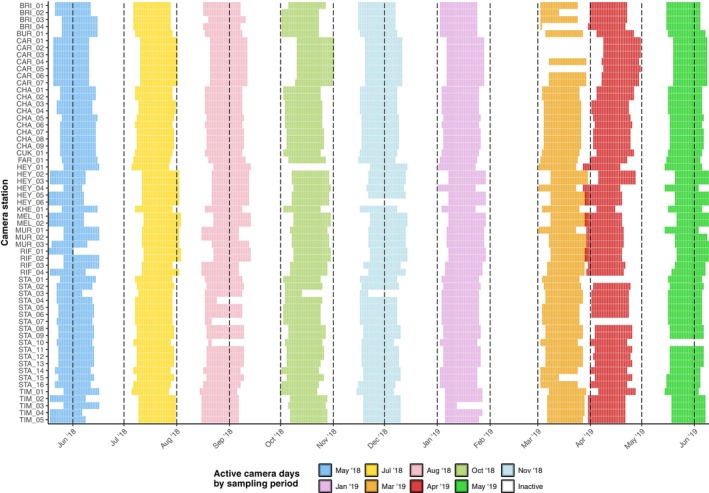
Camera operation chart based on truncated 21‐day sampling period duration. Active camera days are shown by coloured cells. Days on which cameras were inactive (e.g., due to interference, malfunction or battery depletion) or have had data truncated are shown by white cells. Vertical dashed lines show the first day of each calendar month within the survey period.

All camera trap images were processed in digiKam (digiKam Team 2018) by adding species name to tags in the EXIF metadata of each image containing at least one animal. All images were processed by a single researcher (JB) for consistency. The processed data were prepared as a record database in the R package *camtrapR* (Niedballa et al. 2016), using a 15‐minute interval to define independent detection events (Barnes et al. 2025). That is, a detection of a given species was considered independent if it occurred at least 15 minutes after the previous detection of that species. Data from each sampling period were truncated to 21 days to reflect the shortest sampling duration, equating to a total survey effort of 10,966 camera days after correcting for camera failure and malfunction (Table 1; Figure A1). All subsequent analysis used this truncated dataset.

### 2.4 Species Selection

This study focuses on 11 species we have previously identified as rocky landform specialists within the Mount Isa Inlier (Table 2; Barnes et al. 2025). We have considered these species rocky landform specialists in accordance with the definition used by Freeland et al. (1988) of a species with a consistent association with rocky habitats across its entire distribution. We support our classification of each rocky landform specialist through available evidence in the scientific literature.

**TABLE 2:** Eleven rocky landform specialists detected on camera traps in the Mount Isa Inlier and assessed in this study, with references supporting their saxicoline (i.e., rock‐dwelling) habit, and home range estimates for each specialist (or their analogues if no data was available).

Class	Rocky landform specialist	Saxicoline habit	Home range (ha)	Spatial independence assumed at 772 m?	Spatial independence assumed at 3265 m?
Aves (Birds)	Kalkadoon Grasswren *Amytornis ballarae*	Harrington et al. (2017)	Unknown, but maximum reported foraging area for the similarly saxicoline *A. woodwardi* is 12 ha (Noske 1992)	Yes	Yes
	Spinifex Pigeon *Geophaps plumifera leucogaster*	Johnstone (1981)	Unknown. No estimates for similar species available, but *G. plumifera* is considered sedentary (Higgins and Davies 1996)	Likely	Yes
Mammalia	Euro *Osphranter robustus erubescens*	Telfer et al. (2008)	143–223 (Fisher and Owens 2000)	No	Yes
	Purple‐necked Rock‐wallaby *Petrogale purpureicollis*	Eldridge and Johnson (2023)	Unknown. Maximum reported mean home range size for any other *Petrogale* spp. is 170 ha for *P. xanthopus xanthopus* ; however, these were later found to be much smaller following destocking and removal of feral goats (Hayward et al. 2011). Home range sizes for all other reported *Petrogale* spp. < 25 ha (Horsup 1994; Sharp 2009), but long‐range movements of up to 1.5 km to water have been reported (Sharp 2009)	Likely, but with the possibility of occasional long‐range movements	Yes
	Common Rock Rat *Zyzomys argurus*	Woinarski et al. (1992)	0.07–0.12 (Bradley et al. 1988)	Yes	Yes
Reptilia	Slater's Ring‐tailed Dragon *Ctenophorus slateri*	Storr (1967); E. Pianka (2014)	Unknown, but related *C. ornatus* home range < 0.2 ha (Lebas 2001)	Yes	Yes
	Gravelly soil Ctenotus *Ctenotus lateralis*	Storr (1978)	Unknown, but recapture radius for several arid zone *Ctenotus* spp. reported at a maximum of 60 m, indicating likely maximum home range diameter (Read 1998)	Yes	Yes
	Hosmer's Spiny‐tailed Skink *Egernia hosmeri*	Cogger (2018)	Unknown; however, site fidelity is generally high and dispersal low in the rock‐dwelling *Egernia* (Stow and Sunnucks 2004). Even in the large, non‐saxicolous *E. major* , home range is ~1 ha (Osterwalder et al. 2004)	Yes	Yes
	Robust Dtella *Gehyra robusta*	Cogger (2018)	Home range data for geckos not readily available, but maximum 95% utilisation area for arid‐dwelling *Lucasium damaeum* (as *Diplodactylus damaeus*) ~0.2 ha (Henle 1990)	Yes	Yes
	Ridge‐tailed Monitor *Varanus acanthurus*	Cogger (2018)	Unknown, but home range size for arid to semi‐arid *V. gouldii* , and the tropical rock‐dwelling *V. glauerti* and *V. glebopalma* all 7–8 ha (Green and King 1978; Sweet 1999)	Yes	Yes
	Black‐palmed Rock Monitor *Varanus glebopalma*	Sweet (1999)	3.5–7.76 (Sweet 1999)	Yes	Yes

*Note:* The final two columns assess whether camera stations in this study should be considered independent for each species based on home range data at a distance to the nearest neighbouring camera of (a) minimum 772 m and (b) mean 3265 m.

### 2.5 Determining Spatial Independence

To determine spatial independence of cameras for each species, we calculated hypothetical circular home ranges based on the minimum (radius = 386 m) and average (radius = 1632.5 m) distance between adjacent camera stations. This gave a minimum non‐overlapping area of 46.81 ha and an average of 837.25 ha. These values were compared to reported home ranges for all 11 taxa (or their analogues where home range estimates were unavailable) to determine spatial independence (Table 2). We recognise this method cannot account for home range shape or long‐range movements; however, home range data is generally more accessible than movement and dispersal metrics and still provides some context to the likely maximum area covered by an individual regardless of shape. Noting the limitations, our calculation suggests that, on average, camera stations can be considered spatially independent for all 11 specialists (Table 2).

### 2.6 Data Analysis

The prepared record database was used to produce detection histories (occasion length = 1 day) for all 11 taxa in each sampling period using the R package *camtrapR* (Niedballa et al. 2016). Detection histories were analysed in a single‐species, multi‐season occupancy modelling framework (MacKenzie et al. 2003) using the R package *unmarked* (Fiske and Chandler 2011). Four candidate models were produced for each species, assuming constant probability of occupancy, constant extinction probability and constant colonisation probability, and detection probability assumed as constant, or covarying with one or a combination of sampling period and time since baiting (i.e., survey duration; Table 3). Where the best model indicated detection probability covaried with both sampling period and days since baiting, detection probability estimates were compared using conditional inference trees (Hothorn et al. 2006) in the R package *partykit* (Hothorn and Zeileis 2015).

**TABLE 3:** Candidate multi‐season occupancy models assessing factors affecting the detection probability of 11 rocky landform specialists.

Model →	*ψ*(.)*γ*(.)*ε*(.)*p*(.)				*ψ*(.)*γ*(.)*ε*(.)*p*(samp)				*ψ*(.)*γ*(.)*ε*(.)*p*(dsb)				*ψ*(.)*γ*(.)*ε*(.)*p*(samp + dsb)			
Parameter →	*K*	AICc	ΔAICc	LL	*K*	AICc	ΔAICc	LL	*K*	AICc	ΔAICc	LL	*K*	AICc	ΔAICc	LL
*Ab*	4	1355.4	22.22	−673.34	**12**	**1333.18**	**0**	**−651.27**	5	1392.77	59.59	−690.83	13	1351.07	17.89	−658.58
*Gp*	4	173.65	2.58	−82.46	12	185.91	14.85	−77.64	**5**	**171.07**	**0**	**−79.98**	13	184.51	13.44	−75.3
*Or*	4	1848.05	69.39	−919.66	12	1805.89	27.23	−887.62	5	1850.35	71.69	−919.62	**13**	**1778.66**	**0**	**−872.37**
*Pp*	4	5110.01	411.82	−2550.64	12	5054.03	355.84	−2511.7	5	4760.34	62.15	−2374.62	**13**	**4698.19**	**0**	**−2332.14**
*Za*	4	13870.45	1801.04	−6930.86	12	13105.66	1036.25	−6537.51	5	12921.73	852.32	−6455.31	**13**	**12069.41**	**0**	**−6017.75**
*Cs*	4	285.19	8.58	−138.23	12	285.78	9.18	−127.57	**5**	**276.61**	**0**	**−132.75**	13	278.88	2.28	−122.49
*Cl*	4	1028.06	22.84	−509.67	12	1029.82	24.59	−499.59	**5**	**1005.22**	**0**	**−497.06**	13	1007.36	2.14	−486.72
*Eh*	4	2934.56	124.48	−1462.92	12	2881.9	71.82	−1425.63	5	2862.75	52.67	−1425.82	**13**	**2810.08**	**0**	**−1388.08**
*Gr*	4	2145.34	176.12	−1068.3	**12**	**1969.22**	**0**	**−969.29**	5	2117.67	148.45	−1053.28	13	2083.08	113.86	−1024.58
*Va*	4	2154.99	24.82	−1073.13	**12**	**2130.17**	**0**	**−1049.77**	5	2156.32	26.15	−1072.6	13	2132.45	2.28	−1049.27
*Vg*	4	574.16	19.32	−282.71	**12**	**554.83**	**0**	**−262.1**	5	574.06	19.23	−281.48	13	555.99	1.16	−261.04

*Note:* Model terms: *ψ*, occupancy probability; *γ*, colonisation probability; *ε*, extinction probability; *p*, detection probability. Modelled covariates are noted in parentheses after the relevant term. Covariate abbreviations: (.), null; (dsb), days since baiting; (samp), sampling period. Model parameters: ΔAICc, difference in AICc between a model and the preceding model; AICc, Akaike's Information Criteria with small sample correction; *K*, number of estimated parameters for each model; LL, log‐likelihood. For each species, the model with the lowest AICc regardless of ΔAICc was considered the top model (Burnham et al. 2002; Arnold 2010) and is indicated by a highlighted cell and bold type.

Abbreviations: *Ab*, *Amytornis ballarae* ; *Cl*, *Ctenotus lateralis*; *Cs*, *Ctenophorus slateri*; *Eh*, *Egernia hosmeri*; *Gp*, *Geophaps plumifera leucogaster* ; *Gr*, *Gehyra*; *Or*, *Osphranter robustus erubescens*; *Pp*, *Petrogale purpureicollis* ; *Va*, *Varanus acanthurus* ; *Vg*, *Varanus gleobpalma*; *Za*, *Zyzomys argurus* .

In addition to determining when detection probability was highest, we assessed additional parameters to determine optimal survey duration. We used generalised linear models (GLMs; R Core Team 2023) with a Poisson error distribution and a log link function to model days to first detection as a function of the sampling period (fixed effect, categorical covariate). We checked each model for overdispersion and refitted overdispersed models with a quasi‐Poisson error distribution and log link function. We used GLMs in the R package *MASS* (Venables and Ripley 2002) to model detection rate (days detected per 100 camera‐days) as a function of the sampling period (fixed effect, categorical covariate), using a negative binomial error distribution and a log link function to account for overdispersed, zero‐inflated data. Subsequent analysis of deviance tests were conducted on each GLM to determine the significance of the sampling period on the explanatory variable. Where significant, we used Tukey's HSD post hoc test to identify which sampling periods differed, using the *emmeans* R package (Lenth 2023).

The number of days required to be confident of absence was calculated using Kéry's (2002) false absence equation:

$$N_{\min}=\frac{\log\left(\alpha \right)}{\log\;\left(1-p\right)}$$

Here, *N*_min_ is the minimum number of days to infer absence, α is the desired confidence level, and *p* is detection probability. The minimum number of days to infer absence was calculated for each sampling period at confidence levels of 50%, 80%, and 95% from detection probability estimates at 21 days since baiting.

Kernel density estimates of independent detections over the 24‐hour diel cycle were calculated for each sampling period from the record database using the R package *overlap* (Ridout and Linkie 2009). Sunset and sunrise were determined to the nearest 15 minutes on the middle date of each sampling period. The proportion of detections that fell inside and outside this period (i.e., nighttime vs. daytime detections respectively) was calculated in *overlap* to determine the optimum hours for camera operation.

To consider the effect of lunar illumination on detections, we collected post hoc lunar illumination data and assigned it to four levels (0%–25%, 26%–50%, 51%–75% and 76%–100%; fixed effect, categorical covariate) across the survey. Analyses were restricted to taxa with known nocturnal activity (Euro, *Osphranter robustus erubescens*; Purple‐necked Rock‐wallaby, *Petrogale purpureicollis* ; Common Rock Rat, *Zyzomys argurus* and; Robust Dtella, *Gehyra robusta* ). We used generalised linear mixed models (GLMMs) in the R package *lme4* (Bates et al. 2015) to model total detections per 100 camera‐days as a function of lunar illumination, using a negative binomial error distribution and a log link function to account for overdispersed, zero‐inflated data. The response variable was a single‐column matrix of total independent detections by calendar date, illumination level was included as a fixed effect, and sampling period was included as a random effect to control for the influence of sampling period on the number of detections. We subsequently conducted an analysis of variance test on each GLMM to determine the significance of lunar illumination on total detections. Where significant, we used Tukey's HSD post hoc test to identify the lunar illumination scenarios under which total detections differed, using the *emmeans* R package (Lenth 2023).

## 3 Results

### 3.1 Detection Probability

Model selection showed detection probability covaried by sampling period for four species, days since baiting for three species, and both sampling period and days since baiting for four species (Table 3).

#### 3.1.1 Detection Probability Covarying With Sampling Period

**FIGURE 2:**
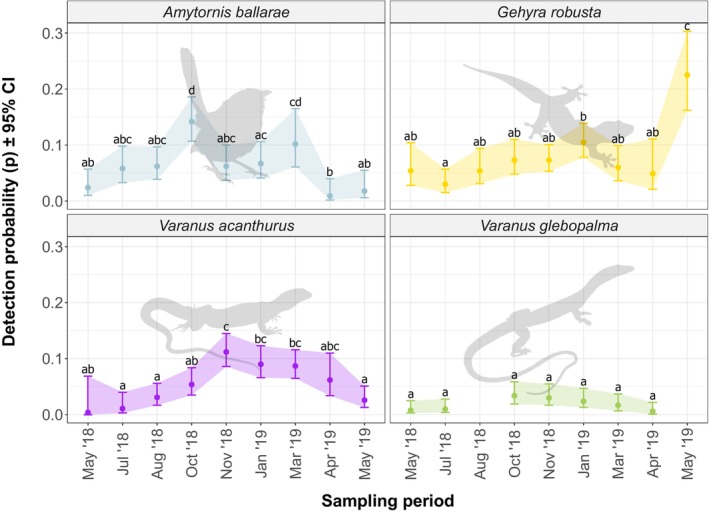
Changes in detection probability (*p*; ±95% CI) between sampling periods for the four rocky landform specialists for which the top occupancy model indicated detection probability covaried with sampling period only. Letters (‘a’, ‘b’, etc.) above error bars indicate Tukey‐style significance groupings. Within each species, sampling periods that share a letter do not have significantly different detection probabilities as judged by 95% CIs.

Detection probability of (a) Kalkadoon Grasswren ( *Amytornis ballarae* ) was significantly higher in October 2018 than all other sampling periods except March 2019; (b) *G. robusta* was significantly higher in May 2019 than all other sampling periods, and higher in January 2019 than July 2018; (c) Ridge‐tailed Monitor ( *Varanus acanthurus* ) was significantly higher in November 2018 than from May to October; and (d) Black‐palmed Rock Monitor ( *V. glebopalma* ) was significantly higher in October 2018 than from May to July (as determined by 95% CIs; Figure 2).

#### 3.1.2 Detection Probability Covarying With Days Since Baiting

**FIGURE 3:**
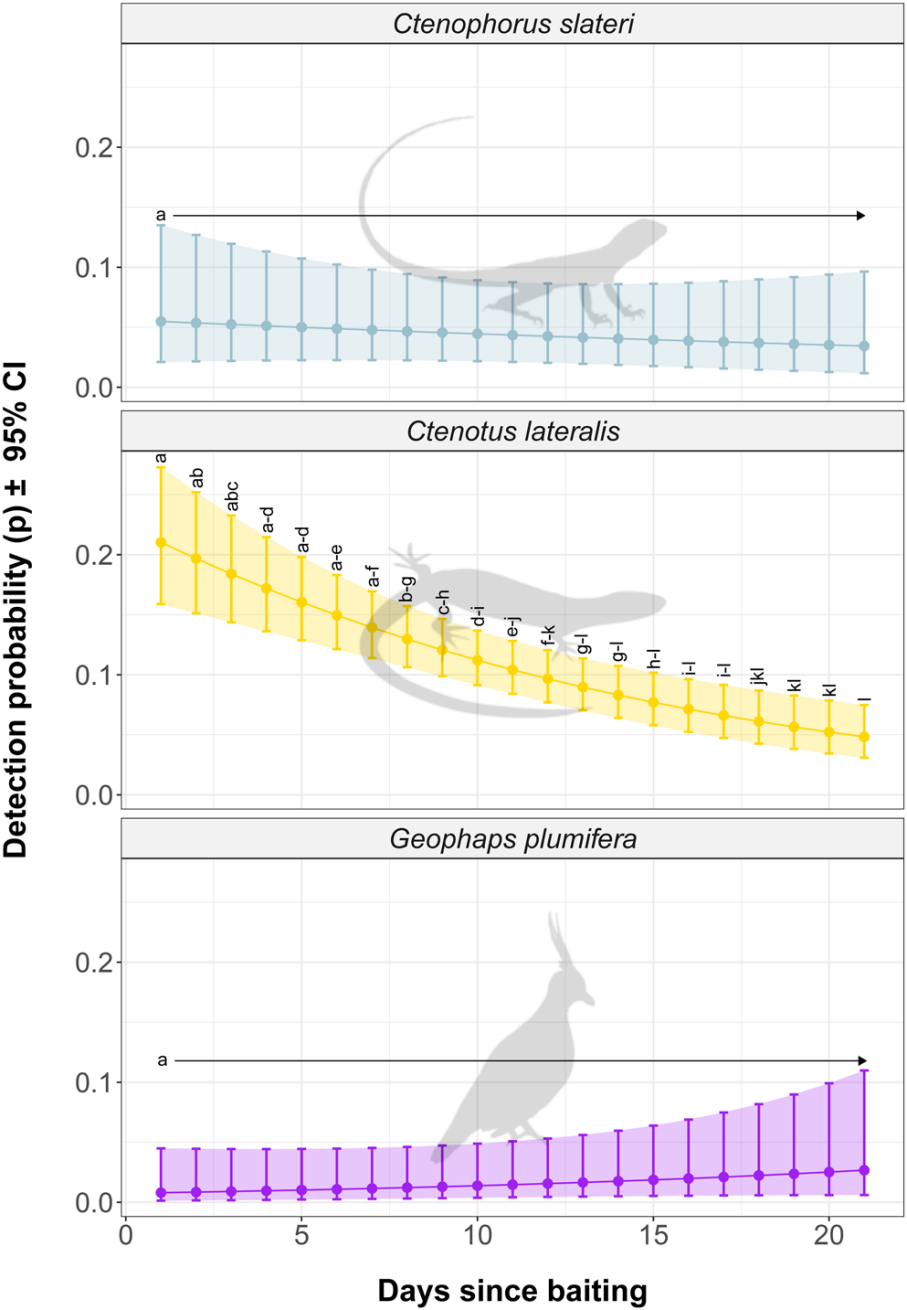
Changes in detection probability (*p*; ±95% CI) over 21 days for the three rocky landform specialists for which the top occupancy model indicated detection probability covaried with days since baiting only. Letters (‘a’, ‘b’, etc.) above error bars indicate Tukey‐style significance groupings. Within each species, days since baiting that share a letter do not have significantly different detection probabilities as judged by 95% CIs.

Detection probability of (a) Slater's Ring‐tailed Dragon (*Ctenophorus slateri*) decreased as days since baiting increased, but was not significantly different at 1 day since baiting than at 21 days; (b) Gravelly soil Ctenotus (*Ctenotus lateralis*) decreased as days since baiting increased and was significantly higher at 1 day since baiting than at 8 days or more; and (c) Spinifex Pigeon ( *Geophaps plumifera leucogaster* ) increased as days since baiting increased but was not significantly different at 1 day since baiting than at 21 days (as determined by 95% CIs; Figure 3).

#### 3.1.3 Detection Probability Covarying With Days Since Baiting and Sampling Period

**FIGURE 4:**
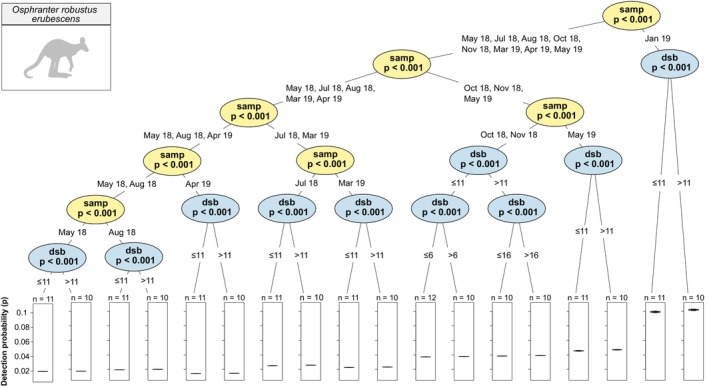
Conditional inference tree of *Osphranter robustus erubescens* detection probability (*p*) estimates by sampling period (‘samp’) and days since baiting (‘dsb’) (±95% CI). At each node, data are partitioned to arrive at a statistically significant binary split. Data on either side of the split are partitioned and split again at the next two nodes. This recursive binary partitioning of data continues until subsequent binary splits are no longer statistically significant. In this tree, the first split partitions sampling period into Jan 19 and all other sampling periods, with detection probability significantly higher in Jan 19. On the following right‐hand branch, the second split partitions days since baiting into ≤ 11 and > 11 days since baiting, with detection probability higher at > 11 days since baiting than at ≤ 11 days since baiting.

**FIGURE 5:**
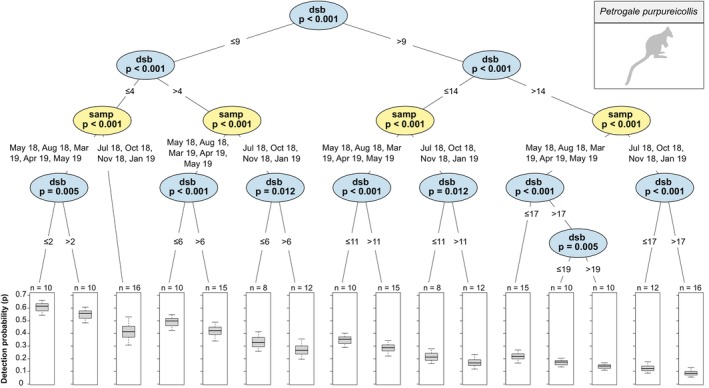
Conditional inference tree of *Petrogale purpureicollis* detection probability (*p*) estimates by sampling period (‘samp’) and days since baiting (‘dsb’). See Figure 4 for an explanation on conditional inference tree interpretation.

**FIGURE 6:**
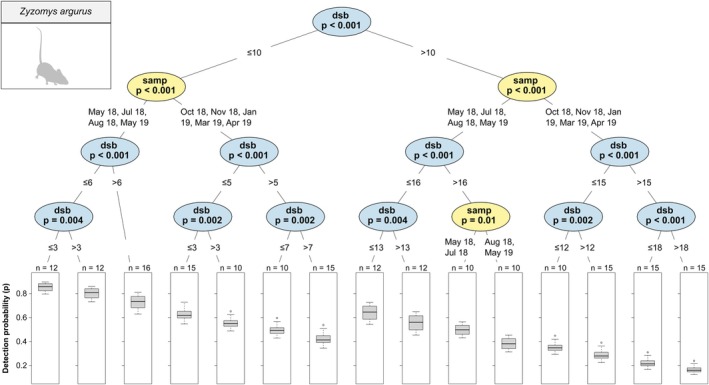
Conditional inference tree of *Zyzomys argurus* detection probability (*p*) estimates by sampling period (‘samp’) and days since baiting (‘dsb’). See Figure 4 for an explanation on conditional inference tree interpretation.

**FIGURE 7:**
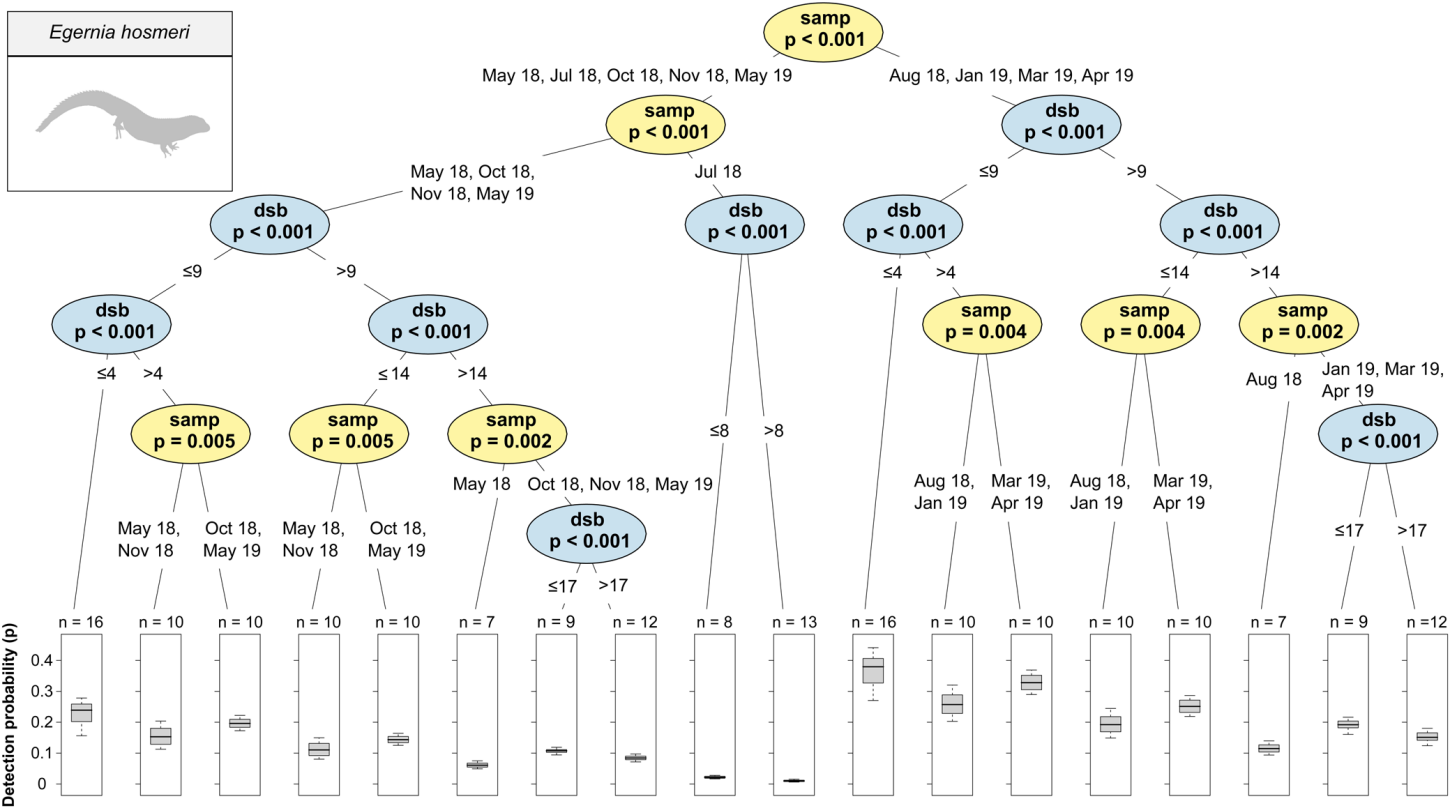
Conditional inference tree of *Egernia hosmeri* detection probability (*p*) estimates by sampling period (‘samp’) and days since baiting (‘dsb’). See Figure 4 for an explanation on conditional inference tree interpretation.

**FIGURE 8:**
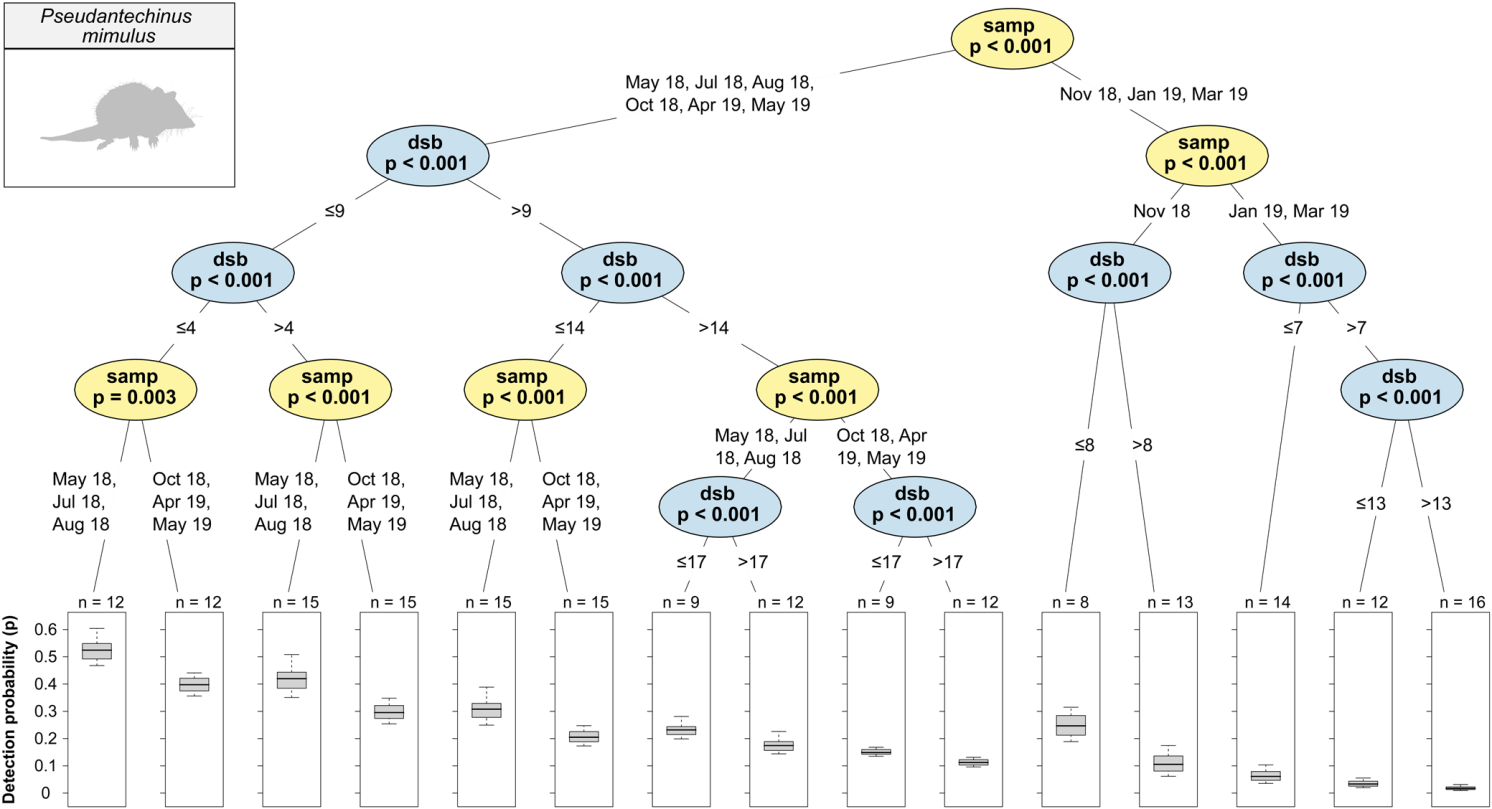
Conditional inference tree of *Pseudantechinus mimulus* detection probability (*p*) estimates by sampling period (‘samp’) and days since baiting (‘dsb’). See Figure 4 for an explanation on conditional inference tree interpretation. See Barnes et al. (2023) for detailed interpretation of this species.

Detection probability of (a) *O. r. erubescens* was significantly higher in January than all other sampling periods and was slightly, but significantly, higher at more than 11 days since baiting than at 11 days or less (Figure 4); (b) *P. purpureicollis* was significantly higher at 9 days or less since baiting than at more than 9 days, regardless of sampling period, and was significantly higher in August and from March to May than all other sampling periods, regardless of days since baiting (Figure 5); (c) *Z. argurus* was significantly higher at 10 days or less since baiting than at 10 days or more, regardless of sampling period, and was significantly higher from May to August than all other sampling periods, regardless of days since baiting (Figure 6) and (d) Hosmer's Spiny‐tailed Skink (*Egernia hosmeri*) was significantly higher in August 2018 and January to March than all other sampling periods and, regardless of sampling period, was higher at 9 days or less since baiting than at 9 days or more (Figure 7). A conditional inference tree for *P. mimulus* is also shown for completeness (Figure 8).

### 3.2 Days to First Detection

**FIGURE 9:**
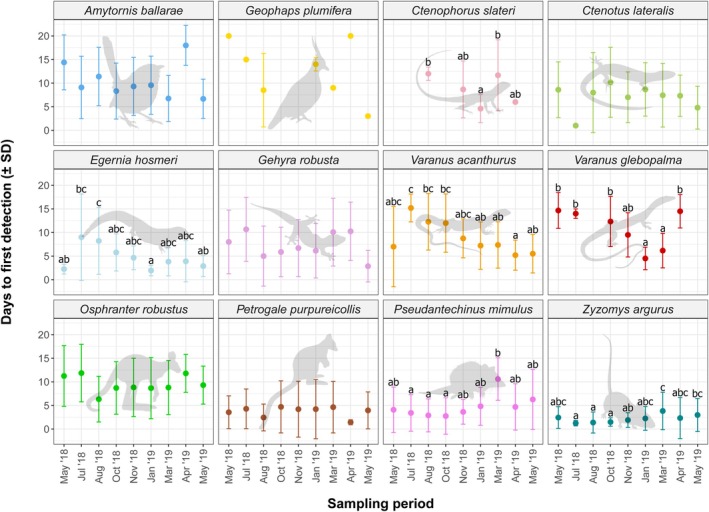
Number of days to first detection (±SD) of rocky landform specialists across all sampling periods. Letters (‘a’, ‘b’, etc.) above error bars indicate Tukey's HSD post hoc test groupings for those species which generalised linear models indicated a significant difference in days to first detection between sampling periods. Within each species, sampling periods that share a letter are not significantly different.

There was a significant effect of sampling period on days to first detection for five species (Table A2). Days to detection was shorter for *Z. argurus* from July to November than from April to May; shorter for *C. slateri* in January than in March and August; shorter for *E. hosmeri* in January than from July to October and shorter in May than in August; shorter for *V. acanthurus* from January to May than in July and shorter in April than from July to August; and shorter for *V. glebopalma* from January to March than from April to July (Figure 9; Table A1; Tukey's HSD).

### 3.3 Detection Rate

**FIGURE 10:**
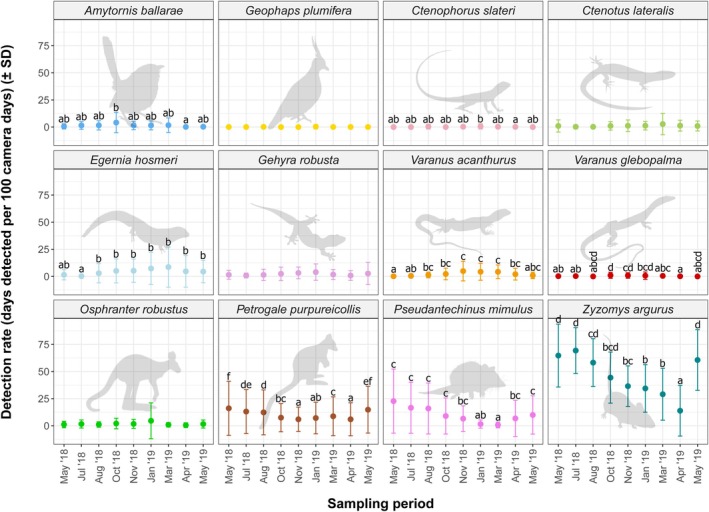
Detection rate (days detected per 100 camera‐days; ±SD) of rocky landform specialists across all sampling periods. Letters (‘a’, ‘b’, etc.) above error bars indicate Tukey's HSD post hoc test groupings for those species which generalised linear models indicated a significant difference in detection rate between sampling periods. Within each species, sampling periods that share a letter are not significantly different.

There was a significant effect of sampling period on detection rate for eight species (Table A3). Detection rate was higher for *A. ballarae* in October than in April; higher for *P. purpureicollis* in May than all other sampling periods; and higher from July to August in all other sampling periods except May; higher for *Z. argurus* from May to July than November to April, and lower in April than all other sampling periods; higher for *C. slateri* in January than April; lower for *E. hosmeri* in July than all other sampling periods; lower for *V. acanthurus* in May 2018 than all other sampling periods except July, and lower in July than from November to March; and higher for *V. glebopalma* from October to January than May to April, and higher from October to November than July (Figure 10; Table A1; Tukey's HSD). Analysis of deviance also indicated a significant effect of sampling period on detection rate of *G. plumifera* , but post hoc analysis was unable to determine the source of this effect.

### 3.4 Determining Absence

**FIGURE 11:**
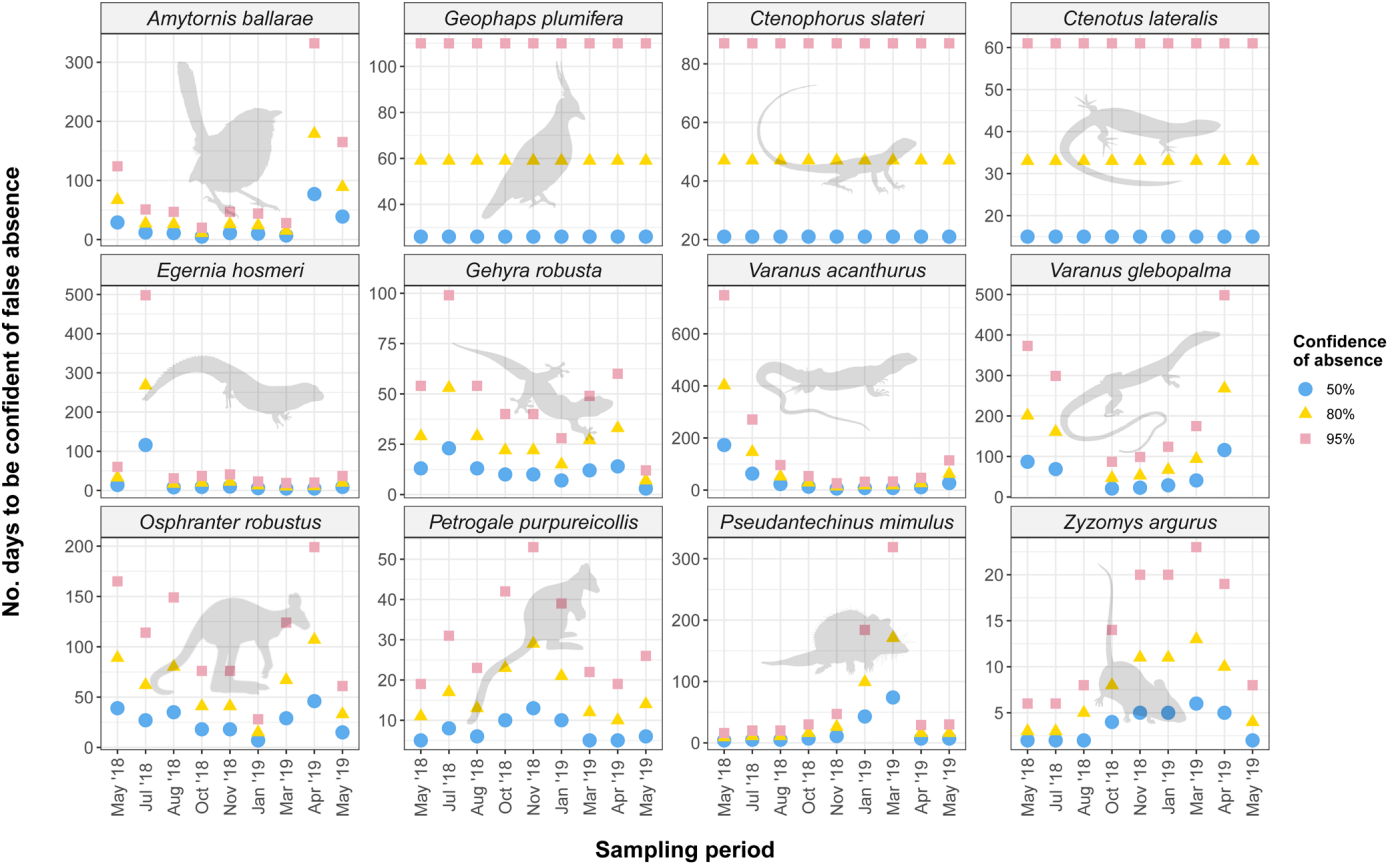
Number of days of survey effort required to be confident of the absence of rocky landform specialists across all sampling periods. Confidence of absence is calculated at three levels (50%, 80% and 95%) using Kéry's (2002) false absence equation, based on detection probability estimates at 21 days. Species with identical estimates across all sampling periods are those for which the top occupancy model indicated no effect of the sampling period on detection probability estimates.

The number of days of effort required to determine absence varies widely by species, sampling period, and desired confidence (Figure 11; Table A1). For instance, attaining a minimum confidence of absence of 80% takes only 3 days for *Z. argurus* in May and July, but takes 59 days for *G. plumifera* across all sampling periods.

### 3.5 Diel Activity

**FIGURE 12:**
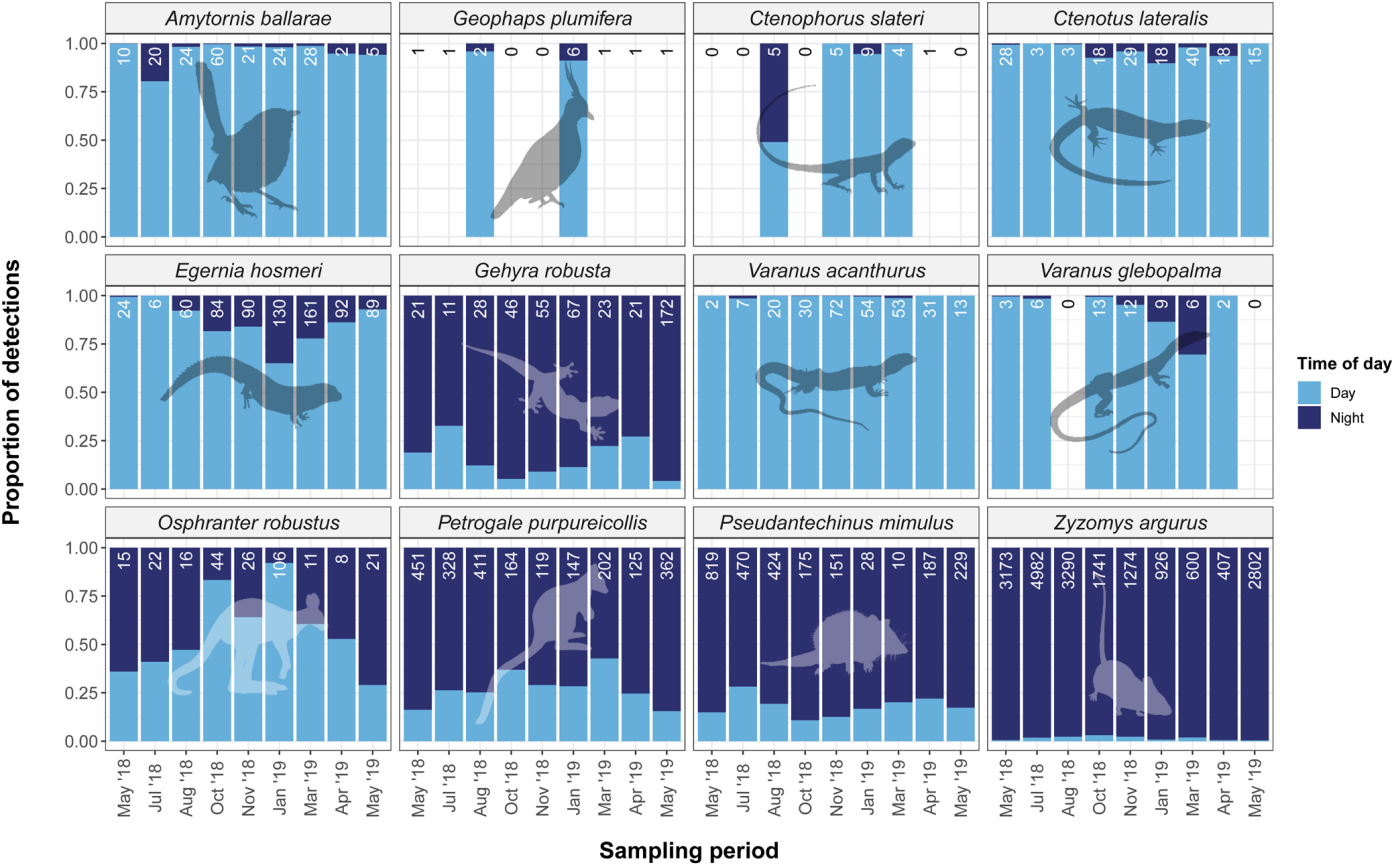
Kernel density estimate‐derived proportion of daytime and nighttime detections of rocky landform specialists across all sampling periods. ‘Day’ refers to detections between sunrise and sunset. ‘Night’ refers to detections between sunset and sunrise. Numbers at the top of each bar are total independent detections over a 21‐day period. No estimates are shown where total detections = 1 as kernel density estimates can only be calculated from two or more observations.

Diel activity varied between species (Figure 12; Table A1). *Amytornis ballarae* , *G. plumifera* , *C. lateralis* , *E. hosmeri*, *V. acanthurus* , and *V. glebopalma* were predominantly diurnally active. *Zyzomys argurus* and *G. robusta* were predominantly nocturnally active. *Osphranter r. erubescens* was more frequently recorded during the day between May and August but still had a relatively high proportion of nocturnal detections. The converse was true between October and April. *Petrogale purpureicollis* was more frequently recorded during the night across the entire survey but still had a moderate proportion of diurnal detections.

### 3.6 Effect of Lunar Illumination

Lunar illumination influenced activity in two species (Table A4). *Zyzomys argurus* had significantly higher detections under 0%–25% illumination than 76%–100% illumination and marginally more detections under 0%–25% illumination than 26%–50% and 51%–75% illumination (Tukey's HSD). *Gehyra robusta* had significantly more detections under 76%–100% illumination than 0%–25% illumination (Tukey's HSD).

## 4 Discussion

This study provides detailed insight into annual changes in detectability of a unique community inhabiting the rocky landforms of the Mount Isa Inlier and demonstrates the applicability of a single‐species camera trap survey to unrelated taxa. Our study presents novel baseline ecological data for many of these taxa and refines existing knowledge for others, revealing distinct temporal patterns in detectability of this community. The remainder of this discussion explores these findings and concludes with recommended camera trap survey guidelines for all taxa.

### 4.1 Detectability of Rocky Landform Specialists

#### 4.1.1 Kalkadoon Grasswren ( *Amytornis ballarae* )

*Amytornis ballarae* is reported to lay from July to October but may raise additional broods throughout the wet season given sufficient rainfall (Higgins et al. 2001). Peaks in detectability for this species in October and March may therefore be a result of increased activity due to higher energy demands during breeding and/or recruitment of juveniles into the population.

#### 4.1.2 Spinifex Pigeon ( *Geophaps plumifera leucogaster* )

*Geophaps p. leucogaster* was ubiquitous in the landscape during this study, but a notable dearth of detections on camera traps suggests camera traps were an inefficient method of detection. Camera traps are rarely used to study birds in Australia (Meek et al. 2015), but can be used effectively for this group, performing well across a range of body sizes (Randler and Kalb 2018; Fontúrbel et al. 2020). Alternatively, because *G. p. leucogaster* infrequently utilises rugged terrain (Barnes et al. 2025), it may be that, on average, camera stations in this study were in less frequented locations. *Geophaps p. leucogaster* is rarely found far from permanent water (Williams et al. 1995; Higgins and Davies 1996), so placement of camera stations on rugged landforms could have been too distant from water to record the species with regularity. Deployment of cameras at or nearer to watering points, and/or baited with a granivore‐specific bait (e.g., Littlewood et al. 2021) may increase detection probability and improve the utility of cameras for this species.

#### 4.1.3 Slater's Ring‐Tailed Dragon (*Ctenophorus slateri*)

Despite frequent observations throughout the study area, *C. slateri* remained rare in camera trap images, suggesting that camera traps were an ineffective survey technique for this species. Small reptiles are less efficiently detected by PIR‐triggered cameras due to their small size and low temperature differential to the surrounding environment (Welbourne 2013; Welbourne et al. 2016). Novel detection methods (e.g., Welbourne 2013) could help compensate for this in future surveys.

#### 4.1.4 Gravelly Soil Ctenotus (*Ctenotus lateralis*)

*Ctenotus lateralis* is a relatively small lizard, smaller even than *C. slateri* (Wilson and Swan 2020) so the relatively high and consistent detection probability of this species was unexpected, and contrasts with findings for arid zone *Ctenotus* spp. which have marked differences in observation rates between months (E. R. Pianka 1969). However, despite our occupancy model estimates, raw camera trap data showed that detections were considerably lower during the July and August 2018 sampling periods (i.e., winter). This is unsurprising, as the likelihood of detecting reptiles increases with temperature (Read and Moseby 2001; Spence‐Bailey et al. 2010). While it is possible that the rocky landform habitat of *C. lateralis* provides a sufficient thermal buffer to permit equal levels of activity throughout the year, we consider it more likely that the overall low number of detections masked the true impact of the winter months on this species.

#### 4.1.5 Hosmer's Spiny‐Tailed Skink (*Egernia hosmeri*)

*Egernia hosmeri* is reported to mate in late August, with young born from December to January (Post 2000 in Chapple 2003). The observed peak in detection probability in August therefore likely corresponds to increased activity due to mating, and the peak from January to April to an influx of juveniles into the population.

This species had a considerable number of nighttime detections in January 2019, coinciding with the highest temperatures recorded during the survey (Bureau of Meteorology 2024). Analysis of kernel density plots (unpub. data) shows these detections mostly occurred within 1–2 hours before sunrise. Since the likelihood of detecting reptiles increases as temperature increases (Read and Moseby 2001; Spence‐Bailey et al. 2010), warm overnight temperatures may have encouraged individuals to begin moving as soon as it was light enough to do so (i.e., before sunrise).

#### 4.1.6 Robust Dtella ( *Gehyra robusta* )

The significantly higher peak in detection probability of *G. robusta* in May 2019 appears to be driven by an increased number of nights on which this species was detected at a single camera station, which post hoc assessment of camera trap imagery indicated was due to the continuous presence of a single animal over multiple nights. We elected not to omit these data but note that this may skew detection probability estimates, and we have interpreted our results accordingly. Noting this, the pattern of detection probability approximates that of annual capture rates for *G. variegata* , which are lowest in late autumn and winter and highest in summer (Bustard 1969). Between years, these data show peaks during the same months but at different rates (Bustard 1969). *Gehyra robusta* may very well follow a similar trend, with the May 2019 peak simply reflecting a shifting baseline.

There was a surprising proportion of daytime detections estimated for this nocturnal species; however, kernel density plots (unpub. data) show these are primarily due to detections around sunrise and sunset. At these times, ambient light may be dim enough for *G. robusta* to emerge from daytime retreats and begin foraging.

*Gehyra robusta* increased activity under high illumination (76%–100%), similar to other gecko species that are reported to increase their activity under high illumination. This is likely due to this group's reliance on visual cues for locating and capturing their arthropod prey, as well as increasing visibility of predators (Spence‐Bailey et al. 2010; Nordberg and Schwarzkopf 2022).

#### 4.1.7 Ridge‐Tailed Monitor ( *Varanus acanthurus* )

*Varanus acanthurus* demonstrated a steady increase in detection probability from May to November, aligning with activity patterns reported for other tropical *Varanus* spp. (Christian et al. 1995, 1996). This coincides with the species' breeding season, which commences in April to May and continues through November (King and Rhodes 1982).

#### 4.1.8 Black‐Palmed Rock Monitor ( *Varanus glebopalma* )

In addition to overall low detections, *V. glebopalma* was never physically observed during the study period. The Mount Isa Inlier is the eastern extent of this species' distribution (Wilson and Swan 2020) and, together with low total detections, this may indicate that the species is naturally rare in the bioregion. Varanids are more effectively recorded using vertically oriented cameras due to their laterally compressed bodies (Moore et al. 2020), so adopting a vertical orientation and using varanid‐specific bait (e.g., Moore et al. 2020) may improve detection probability estimates.

A relatively high proportion of nighttime detections was estimated for *V. glebopalma* in March 2019, but the species was predominantly recorded during the daytime throughout the rest of the year, contrasting with previous work indicating it is a frequent nocturnal forager (Rhind et al. 2013). While it is difficult to draw strong conclusions from our data due to the small number of detections, the pattern we observed may be due to prey‐switching behaviour. *Varanus glebopalma* specialises in lizard prey and most known prey items are diurnally active (James et al. 1992); however, Sweet (1999) documented a dietary shift from lizards to grasshoppers between April and July. Increased nighttime detections during the March 2019 sampling period may reflect a corresponding shift in foraging strategy and prey activity patterns.

#### 4.1.9 Euro (*Osphranter robustus erubescens*)

Regardless of time since baiting, *O. r. erubescens* had significantly higher detection probability in January 2019 than all other sampling periods. Aboriginal Australian knowledge from Arnhem Land suggests that *O. r. erubescens* is more adaptable than other rock‐dwelling macropods and equally utilises lower‐lying areas and rocky habitat except during the wet season, when it utilises rocky habitat with greater frequency (Telfer and Garde 2006). Our results appear to support this knowledge, showing a considerable increase in detection probability at the peak of the wet season; however, raw camera trap data showed that three camera stations accounted for ~88% of all detections of this species during this sampling period. These stations all included potential daytime shelter (e.g., rocky overhangs), which may also explain the high proportion of daytime detections in January 2019. Although these stations may have biased detection probability estimates, this result could also indicate that the presence of shelter sites on rocky terrain is a major driver for increased use of such areas by this species.

#### 4.1.10 Purple‐Necked Rock‐Wallaby ( *Petrogale purpureicollis* )

The observed changes in detection probability across the year for *P. purpureicollis* largely align with that reported for *P. lateralis* (Pentland 2014), with decreased detectability over spring and summer compared to autumn and winter, with the exception of July 2018, which corresponded with the lowest temperatures recorded during the survey (Bureau of Meteorology 2024). This likely resulted in reduced activity (and therefore decreased detection probability) as reported for *P. lateralis* (Pentland 2014).

#### 4.1.11 Common Rock Rat ( *Zyzomys argurus* )

*Zyzomys argurus* had significantly lower detection probability between October and April. Previous population studies on the species (Begg 1981; Bradley et al. 1988) have shown that detectability and trappability of this species are significantly lower in April, likely due to mortality (Bradley et al. 1988) and reduced mobility during the reproductive period (Begg 1981). While our data support this, showing a considerably lower detection rate in April than other sampling periods, it additionally suggests that the species becomes less detectable much earlier in the year than previously suggested, with significantly lower detection probability throughout the wet season.

This species increased activity under low illumination (0%–25%). In general, rodents suppress activity under bright moonlight (Prugh and Golden 2014), and our results suggest that *Z. argurus* conforms to this pattern.

Although the results presented here appear to show a seasonal trend in detection probability for most species, our study only represents a single 13‐month period, and the observed patterns may not hold under all scenarios. Differences between years may occur due to one or more potentially interacting phenomena such as changed environmental conditions and their flow‐on effects (e.g., increased rainfall stimulating changes in primary production), natural die‐off of short‐lived species, predation, immigration and emigration, or loss of a novelty effect from bait. Future research, particularly conducted over multiple years, should incorporate site‐ and year‐level predictors to investigate changes in detection probability between years.

### 4.2 Recommendations for Optimised Camera Trap Surveys of Rocky Landform Specialists of the Mount Isa Inlier

The remainder of this discussion defines optimal camera trap parameters for rocky landform specialists and provides recommended camera trap survey guidelines for all specialists, including our findings on *P. mimulus* (Barnes et al. 2023). These guidelines are summarized in Table 4, presenting recommended timing, duration, and other key parameters as a practical reference for survey planning. The rationale behind these recommendations is discussed in detail in the following sections.

**TABLE 4:** Recommendations for optimised camera trap surveys for 12 rocky landform specialists of the Mount Isa Inlier.

Rocky landform specialist		Optimum survey month(s) and duration (days)^a^												Min. survey duration (days)^b^	Hours of operation		Flash type		Lunar illumination (%)				Baiting	
		Jan^a^	Feb	Mar^a^	Apr^a^	May^a^, ^c^	Jun	Jul^a^	Aug^a^	Sep	Oct^a^	Nov^a^	Dec		Day	Night	Black	White	0–25	26–50	51–75	76–100	PB	Alt.
Aves (Birds)	*Ab*					15					11			NA	●		●	●	NA					●
	*Gp* ^d^	59												NA	●		●	●	NA					●
Mammalia	*Or*	15												11	●	●	●	●	●	●	●	●		●
	*Pp*			12	10	11			13					2	●	●	●	●	●	●	●	●	●	
	*Pm*				16	9		11	11		16			4	●	●		●	●	●	●	●	●	
	*Za*					3		3	5					3		●		●	●	○	○		●	
Reptilia	*Cs* ^d^	47												NA	●		●	●	NA					●
	*Cl*	33												7	●		●	●	NA				●	
	*Eh*	13		10	11				17					4	●		●	●	NA				●	
	*Gr* ^e^	15	27	33		29			29		22	22		NA		●		●		●	●	●		●
	*Va*	18		18								14		NA	●		●	●	NA					●
	*Vg* ^d^	124		175							87	99		NA	●		●	●	NA					●

*Note:* (●) indicate parameters that are suitable for a given species; (○) indicate parameters that are marginally suitable for a given species.

Abbreviations: Rocky landform specialists: *Ab*, *Amytornis ballarae* ; *Cl*, *Ctenotus lateralis*; *Cs*, *Ctenophorus slateri*; *Eh*, *Egernia hosmeri*; *Gp*, *Geophaps plumifera leucogaster* ; *Gr*, *Gehyra*; *Or*, *Osphranter robustus erubescens*; *Pm*, *Pseudantechinus mimulus* ; *Pp*, *Petrogale purpureicollis* ; *Va*, *Varanus acanthurus* ; *Vg*, *Varanus gleobpalma*; *Za*, *Zyzomys argurus* . Baiting: Alt, explore alternatives; PB, peanut butter.

^a^ Indicates months in which sampling was conducted. Grey‐shaded cells indicate optimum survey month(s) for each species and numbers in each grey‐shaded cell indicate optimum survey duration (days).

^b^ Minimum duration is only given for species whose occupancy models included ‘days since baiting’.

^c^ Sampling in May was conducted in 2018 and 2019—survey duration is based on whichever of the two sampling periods had lower detection probability, with the recognition that required duration may vary between years.

^d^ These species were not readily detected on camera traps and required survey durations to achieve 80% confidence of absence may be prohibitive.

^e^ Excludes the May 2019 data due to anomalously high number of detections at a single camera station, leading to heavily skewed detection probability estimates.

Although this study provides insights into all specialists assessed, we reiterate that strong conclusions cannot be drawn for all of them. *Ctenophorus slateri*, *V. glebopalma*, and *G. p. leucogaster* all had insufficient detections to confidently determine annual patterns, with each being absent from camera trap images during at least one sampling period. Provisional recommendations are made for these species based on the data available; however, given the temporal gaps in the data, these recommendations should be considered in the broader context of this discussion.

All detection probability estimates and derived parameters, especially survey duration, are based on our use of an array of 60 cameras; however, this may be unrealistic for some practitioners. Because detection probability can only be calculated from occupied sites (Mackenzie 2005), using fewer cameras will lead to lower detection probability estimates due to having fewer sites on which to calculate these estimates. In these instances, the guidelines in Table 4 still provide valuable information on survey timing, but increases to survey duration may be required, especially if determining absence is a key aim.

#### 4.2.1 Time of Year

Sampling limitations mean some months are extrapolated as suitable based on adjacent data (e.g., if November and January are suitable, December is assumed to be), though intermediate conditions may affect detection probability. Similarly, unsampled months may be suitable but unrecognised as such. While our current dataset cannot resolve these gaps, future surveys could address them by sampling year round.

#### 4.2.2 Survey Duration

Recommended survey durations are principally determined on achieving a confidence of absence of 80%. While it would be preferable to conduct surveys to achieve 95% confidence, the required duration is likely to be unreasonable for most species and survey scenarios. Duration is determined on a detection probability point estimate at 21 days as these generally provide the narrowest confidence intervals and therefore the most accurate recommendations. For consistency, especially over long survey periods, it may be preferable to align all survey periods to the longest recommended duration (e.g., 5 days for *Z. argurus* ). There is no great disadvantage to extending camera operation over the minimum duration required to maximise detection probability, and doing so may alleviate concerns about potential changes in detection probability between years; however, this is at the discretion of the practitioner. We expect our guidelines to be implemented in one of two scenarios: (1) if presence of a species at a site is unknown and the primary focus is therefore determining absence to a desired confidence level at a given site; or (2) species presence is previously known from a site but it is desirable to determine whether the species persists there (e.g., for long‐term monitoring).

#### 4.2.3 Hours of Operation

Hours of operation are specified as ‘Daytime only’, ‘Nighttime only’ or ‘Daytime and Nighttime’ and reflect when cameras should be active for targeting a given species. These recommendations relate specifically to the proportion of daytime versus nighttime detections at the times of year where detection probability is maximised. Setting cameras to operate for only part of the diel cycle has two key advantages, particularly for nighttime‐only operation: (1) reduced battery consumption (reduced likelihood of camera failure) and; (2) a reduced number of photos (reduced post‐survey time and cost). Regardless of our recommendations, there are scenarios in which 24‐hour operation may be desirable (e.g., exploring diel activity patterns). Such decisions are context‐specific and at the discretion of the practitioner.

#### 4.2.4 Flash Type

A recommendation for nighttime operation assumes white‐flash cameras. Despite the possibility of aversion to white‐flash for some nocturnal taxa (e.g., Wegge et al. 2004; Schipper 2007; Glen et al. 2013), camera trap imagery does not support this for nocturnally active rocky landform specialists detected during this project. *Osphranter r. erubescens*, *P*. *purpurecollis*, and *Z. argurus* rarely appeared startled and were frequently recorded in front of cameras at night for multiple triggers. Individuals of these species could also at least sometimes be distinguished due to characteristic morphology (e.g., scars, cropped tails, colouration) and were observed returning to camera stations on multiple occasions. Particularly for small mammals within the study area, white‐flash cameras are preferable due to the presence of a range of syntopic species that may be difficult to distinguish in black‐and‐white images (e.g., *Planigale* sp., *Pseudantechinus mimulus* , *Pseudomys desertor* , *Zyzomys argurus* ), especially if part of the animal is obscured. White‐flash cameras are not strictly necessary for large and/or diurnal species. If surveys are intended to only target such species, black‐flash cameras could be used if desired (e.g., due to cost or availability).

#### 4.2.5 Lunar Illumination

Recommendations for conducting surveys under varying lunar illumination scenarios are provided for species that are at least partially nocturnal, although only two demonstrated an effect of lunar illumination on activity levels. For ease of analysis, lunar illumination was considered in equal quartiles spanning 0%–100% illumination, which may obscure some fine‐scale inferences. For example, *Z. argurus* had a significantly higher number of detections under 0%–25% illumination, but there is no way of determining if there was a difference between 0% (i.e., new moon) and 25% illumination.

#### 4.2.6 Baiting

Baiting recommendations are given as either ‘PB’ or ‘Explore alternatives’. ‘PB’ refers specifically to the appropriateness of peanut butter bait and is based on two conditions being satisfied: (1) an occupancy model including ‘days since baiting’ and showing a significant decrease in detection probability after several days and; (2) observed consumption of bait by a species in camera trap images. For species attracted to a given bait, camera trap data tends to demonstrate a pulse of activity immediately following baiting, with a subsequent reduction in activity until bait is replenished (Paull et al. 2011; Weerakoon and Ruffino 2014; Smith et al. 2017; Barnes et al. 2023), and initial peaks in detection probability for several specialists likely reflects this phenomenon. For species where there was no effect of ‘days since baiting’ on detection probability or where bait consumption was never observed, peanut butter bait is assumed to be unsuitable. While our results indicate unbaited cameras would have been as effective as baited cameras for detecting these species throughout this project, we make the recommendation for ‘Explore alternatives’, rather than ‘No bait’. There is no universal bait, with different bait types having variable effectiveness on different species (Davis et al. 2008; Paull et al. 2011; Avrin et al. 2021; Mortelliti et al. 2024), so exploration of alternatives may improve detection probability estimates for some species.

## Author Contributions

**Jarrad C. Barnes:** conceptualization (lead), data curation (lead), formal analysis (lead), investigation (lead), methodology (lead), writing – original draft (lead). **Elizabeth A. Brunton:** supervision (equal), writing – review and editing (equal). **Mark G. Sanders:** funding acquisition (lead), supervision (supporting), writing – review and editing (equal). **Christofer J. Clemente:** supervision (equal), writing – review and editing (equal).

## Conflicts of Interest

The authors declare no conflicts of interest.

## Data Availability

The data that support the findings of this study are openly available on the University of the Sunshine Coast Research Bank at https://doi.org/10.25907/00914.

## Appendix A

**TABLE A1:** Additional parameters affecting detectability of rocky landform specialists.

Species	Sampling period	Detection rate (nights detected/100 trap‐nights) (±SD)	Days to first detection (±SD)	Proportion of detections		Confidence of absence		
				Day	Night	50%	80%	95%
*Amytornis ballarae* Kalkadoon Grasswren	May '18	0.63 ± 2.4	14.4 ± 5.81	0.998	0.002	29	67	124
	July '18	1.51 ± 3.56	9.09 ± 6.61	0.804	0.196	12	27	51
	August '18	1.59 ± 4.27	11.4 ± 6.19	0.982	0.018	11	26	47
	October '18	4.14 ± 9.36	8.31 ± 5.93	0.996	0.004	5	11	20
	November '18	1.54 ± 3.9	9.3 ± 6.15	0.984	0.016	11	26	47
	January '19	1.52 ± 4.1	9.56 ± 6.17	0.98	0.02	10	24	44
	March '19	1.7 ± 6.76	6.75 ± 4.89	0.987	0.013	7	15	28
	April '19	0.16 ± 0.88	18 ± 4.24	0.947	0.053	77	179	332
	May '19	0.32 ± 1.5	6.67 ± 4.16	0.941	0.059	39	89	165
*Geophaps plumifera leucogaster* Spinifex Pigeon	May '18	0.08 ± 0.61	20 ± 0	0	0	26	59	110
	July '18	0.08 ± 0.61	15 ± 0	0	0			
	August '18	0.16 ± 0.86	8.5 ± 7.78	0.958	0.042			
	October '18	0	0	0	0			
	November '18	0	0	0	0			
	January '19	0.32 ± 1.93	14 ± 1.41	0.911	0.089			
	March '19	0.09 ± 0.64	9 ± 0	0	0			
	April '19	0.08 ± 0.63	20 ± 0	0	0			
	May '19	0.08 ± 0.62	3 ± 0	0	0			
*Osphranter robustus erubescens* Euro	May '18	1.19 ± 2.99	11.25 ± 6.44	0.36	0.64	39	89	165
	July '18	1.67 ± 3.7	11.86 ± 6.09	0.41	0.59	27	62	114
	August '18	1.19 ± 2.57	6.33 ± 4.83	0.472	0.528	35	80	149
	October '18	2.11 ± 4.84	8.71 ± 5.57	0.833	0.167	18	41	76
	November '18	1.78 ± 4.14	8.83 ± 6.18	0.641	0.359	18	41	76
	January '19	4.6 ± 16.52	8.67 ± 6.47	0.921	0.079	7	15	28
	March '19	0.94 ± 2.11	8.8 ± 5.73	0.605	0.395	29	67	124
	April '19	0.57 ± 2.2	11.8 ± 4.02	0.528	0.472	46	107	199
	May '19	1.62 ± 3.71	9.31 ± 4.03	0.29	0.71	15	33	61
*Petrogale purpureicollis* Purple‐necked Rock‐wallaby	May '18	16.12 ± 24.94	3.56 ± 3.47	0.162	0.838	5	11	19
	July '18	13.12 ± 20.28	4.29 ± 4.2	0.263	0.737	8	17	31
	August '18	12.38 ± 20.7	2.45 ± 2.84	0.252	0.748	6	13	23
	October '18	7.51 ± 13.03	4.7 ± 5.52	0.369	0.631	10	23	42
	November '18	5.99 ± 11.23	4.21 ± 5.91	0.29	0.71	13	29	53
	January '19	7.16 ± 14.57	4.22 ± 6.27	0.284	0.716	10	21	39
	March '19	8.79 ± 17.94	4.64 ± 5.46	0.427	0.573	5	12	22
	April '19	5.99 ± 15.23	1.44 ± 0.53	0.246	0.754	5	10	19
	May '19	14.85 ± 21.55	3.96 ± 3.91	0.155	0.845	6	14	26
*Pseudantechinus mimulus* Carpentarian Pseudantechinus	May '18	22.75 ± 29.53	4.09 ± 4.81	0.149	0.851	4	9	16
	July '18	16.63 ± 23.54	3.42 ± 3.87	0.282	0.718	5	11	20
	August '18	15.95 ± 23.49	2.9 ± 3.5	0.193	0.807	5	11	20
	October '18	9.03 ± 16.6	2.76 ± 3.86	0.108	0.892	7	16	30
	November '18	6.57 ± 11.85	3.64 ± 2.61	0.126	0.874	11	26	47
	January '19	1.59 ± 3.79	4.83 ± 4.02	0.167	0.833	43	99	184
	March '19	0.77 ± 2.69	10.6 ± 4.51	0.201	0.799	74	171	319
	April '19	6.81 ± 16.69	4.69 ± 4.91	0.22	0.78	7	16	29
	May '19	9.98 ± 17.69	6.28 ± 6.36	0.173	0.827	7	16	30
*Zyzomys argurus* Common Rock Rat	May '18	64.73 ± 28.98	2.44 ± 2.31	0.005	0.995	2	3	6
	July '18	69.35 ± 21.13	1.25 ± 0.57	0.018	0.982	2	3	6
	August '18	58.25 ± 22.04	1.39 ± 2.23	0.023	0.977	2	5	8
	October '18	44.42 ± 23.53	1.49 ± 0.89	0.031	0.969	4	8	14
	November '18	36.53 ± 18.72	1.91 ± 1.56	0.023	0.977	5	11	20
	January '19	34.44 ± 21.93	2.25 ± 2.52	0.008	0.992	5	11	20
	March '19	29.08 ± 23.92	3.83 ± 3.97	0.019	0.981	6	13	23
	April '19	13.88 ± 23.35	2.33 ± 4.35	0.005	0.995	5	10	19
	May '19	60.64 ± 27.99	2.98 ± 3.49	0.004	0.996	2	4	8
*Ctenophorus slateri* Slater's Ring‐tailed Dragon	May '18	0	0	0	0	21	47	87
	July '18	0	0	0	0			
	August '18	0.4 ± 2.2	12 ± 1.41	0.49	0.51			
	October '18	0	0	0	0			
	November '18	0.32 ± 1.5	8.67 ± 6.03	1	0			
	January '19	0.63 ± 2.4	4.6 ± 2.97	0.945	0.055			
	March '19	0.26 ± 1.08	11.67 ± 7.51	0.997	0.003			
	April '19	0.16 ± 1.19	6 ± 0	0	0			
	May '19	0	0	0	0			
*Ctenotus lateralis* Gravelly soil Ctenotus	May '18	1.03 ± 5.62	8.6 ± 5.9	0.993	0.007	15	33	61
	July '18	0.24 ± 1.84	1 ± 0	1	0			
	August '18	0.24 ± 1.37	8 ± 8.49	0.995	0.005			
	October '18	1.06 ± 3.88	10.17 ± 7.41	0.925	0.075			
	November '18	1.21 ± 5.35	7 ± 5.37	0.958	0.042			
	January '19	1.27 ± 3.91	8.67 ± 5.66	0.897	0.103			
	March '19	2.72 ± 9.82	7.43 ± 6.73	0.979	0.021			
	April '19	1.31 ± 5.03	7.33 ± 4.37	0.935	0.065			
	May '19	0.98 ± 4.58	4.8 ± 4.55	1	0			
*Egernia hosmeri* Hosmer's Spiny‐tailed Skink	May '18	1.51 ± 4.68	2.25 ± 1.04	0.993	0.007	14	33	60
	July '18	0.24 ± 1.05	9 ± 9.17	1	0	116	268	498
	August '18	2.94 ± 8.86	8.22 ± 7.08	0.922	0.078	8	17	31
	October '18	5.08 ± 11.04	5.78 ± 3.93	0.816	0.184	9	20	37
	November '18	5.26 ± 10.75	4.65 ± 2.52	0.839	0.161	10	22	41
	January '19	7.3 ± 14.74	1.94 ± 1.11	0.65	0.35	6	13	23
	March '19	8.65 ± 18.56	3.82 ± 3	0.778	0.222	5	10	19
	April '19	4.68 ± 14.56	3.9 ± 4.36	0.862	0.138	5	11	20
	May '19	4.47 ± 10.33	2.88 ± 2.22	0.928	0.072	9	20	37
*Gehyra robusta* Robust Dtella	May '18	1.49 ± 4.03	8 ± 6.75	0.189	0.811	13	29	54
	July '18	0.87 ± 2.23	10.67 ± 6.78	0.327	0.673	23	53	99
	August '18	1.43 ± 5.21	5 ± 6.36	0.122	0.878	13	29	54
	October '18	2.42 ± 6.21	5.87 ± 5.25	0.052	0.948	10	22	40
	November '18	3.23 ± 5.41	6.7 ± 6.05	0.09	0.91	10	22	40
	January '19	3.97 ± 7.55	6.14 ± 5.77	0.113	0.887	7	15	28
	March '19	1.78 ± 4.58	10.08 ± 7.18	0.222	0.778	12	27	49
	April '19	0.82 ± 4.47	10.25 ± 6.18	0.271	0.729	14	33	60
	May '19	2.67 ± 10.21	2.86 ± 3.34	0.042	0.958	3	7	12
*Varanus acanthurus* Ridge‐tailed Monitor	May '18	0.16 ± 0.86	7 ± 8.49	1	0	173	402	748
	July '18	0.48 ± 1.69	15.2 ± 2.95	0.985	0.015	63	146	271
	August '18	1.35 ± 2.64	12.27 ± 5.99	0.999	0.001	23	52	96
	October '18	2.26 ± 5.33	12 ± 6.18	0.997	0.003	13	29	54
	November '18	4.92 ± 9.06	8.75 ± 4.11	0.998	0.002	6	14	26
	January '19	4.29 ± 7.83	7.22 ± 5.02	0.995	0.005	8	18	32
	March '19	4.25 ± 6.33	7.36 ± 4.91	0.987	0.013	8	18	33
	April '19	2.05 ± 5.36	5.2 ± 3.19	1	0	11	26	47
	May '19	0.97 ± 3.03	5.5 ± 4.07	1	0	27	62	114
*Varanus glebopalma* Black‐palmed Rock Monitor	May '18	0.24 ± 1.05	14.67 ± 3.79	0.995	0.005	87	201	373
	July '18	0.32 ± 1.48	14 ± 1	0.984	0.016	69	161	299
	August '18	0	0	0	0	—	—	—
	October '18	1.05 ± 2.81	12.33 ± 5.32	0.993	0.007	21	47	87
	November '18	0.89 ± 2.57	9.5 ± 4.69	0.952	0.048	23	53	99
	January '19	0.71 ± 3.38	4.5 ± 2.38	0.864	0.136	29	67	124
	March '19	0.51 ± 1.49	6.17 ± 3.66	0.695	0.305	41	94	175
	April '19	0.16 ± 0.88	14.5 ± 3.54	1	0	116	268	498
	May '19	0	0	0	0	—	—	—

*Note:* ‘Proportion of detections’ are derived from kernel density estimates based on sunset and sunrise times of the middle date of each sampling period. Confidence of absence indicates the number of days required to achieve confidence that a species is absent at confidence levels of 50%, 80% and 95% (Kéry 2002).

**TABLE A2:** Results of analysis of deviance tests on generalised linear models (GLMs) comparing days to first detection for all rocky landform specialists between all sampling periods.

Species	DF	Deviance	Residual DF	Residual deviance	*F*	*p*
*Amytornis ballarae*	8	38.09	65	260.51	1.27	0.28
*Geophaps plumifera*	6	22.83	2	7.87	1.05	0.56
*Osphranter robustus*	8	31.06	95	350.76	1.12	0.36
*Petrogale purpureicollis*	8	39.47	174	689.55	0.97	0.46
***Zyzomys argurus***	**8**	**129.7**	**462**	**724.34**	**6.32**	**< 0.001**
***Ctnophorus slateri***	**4**	**17.09**	**9**	**28.53**	**NA**	**0.002**
*Ctenotus lateralis*	8	22.08	38	184.82	0.62	0.76
***Egernia hosmeri***	**8**	**94.21**	**110**	**254.11**	**4.98**	**< 0.001**
*Gehyra robusta*	8	69.99	103	519.77	1.64	0.12
***Varanus acanthurus***	**8**	**96.73**	**119**	**357.72**	**4.24**	**< 0.001**
***Varanus glebopalma***	**8**	**43.62**	**28**	**57.97**	**NA**	**< 0.001**

*Note:* GLMs used a quasi‐Poisson error rate to account for overdispersion, except those where *F* = NA. Models where *F* = NA indicate no overdispersion and therefore the use of a Poisson error rate and subsequent chi‐squared test. Species for which tests were significant (*p* < 0.05) are highlighted in bold.

**TABLE A3:** Results of analysis of deviance tests on negative binomial generalised linear models comparing detection rate (days detected per 100 camera‐days) for all rocky landform specialists between all sampling periods.

Species	DF	Deviance	Residual DF	Residual deviance	*p*
***Amytornis ballarae***	**8**	**17.53**	**522**	**173.53**	**0.025**
***Geophaps plumifera***	**8**	**44.37**	**522**	**393.14**	**< 0.001**
*Osphranter robustus*	8	12.03	522	235.61	0.15
***Petrogale purpureicollis***	**8**	**714.05**	**522**	**13,568**	**< 0.001**
***Zyzomys argurus***	**8**	**134.82**	**522**	**669.69**	**< 0.001**
***Ctnophorus slateri***	**8**	**145.11**	**522**	**634.17**	**< 0.001**
*Ctenotus lateralis*	8	6.42	522	111.48	0.601
***Egernia hosmeri***	**8**	**23.53**	**522**	**257.11**	**0.003**
*Gehyra robusta*	8	10.3	522	249.72	0.245
***Varanus acanthurus***	**8**	**45.93**	**522**	**286.5**	**< 0.001**
***Varanus glebopalma***	**8**	**192.39**	**522**	**1111.8**	**< 0.001**

*Note:* Species for which tests were significant (*p* < 0.05) are highlighted in bold.

**TABLE A4:** Results of analysis of variance tests on negative binomial generalised linear mixed models (GLMMs) testing for an effect of lunar illumination on detections (total independent detections per 100 camera‐days) of rocky landform specialists across all sampling periods.

Species	DF	Chisq	*p*
*Amytornis ballarae*	NA		
*Geophaps plumifera*	NA		
*Osphranter robustus*	3	6.66	0.084
*Petrogale purpureicollis*	3	2.31	0.51
***Zyzomys argurus***	**3**	**13.83**	**0.003**
*Ctnophorus slateri*	NA		
*Ctenotus lateralis*	NA		
*Egernia hosmeri*	NA		
***Gehyra robusta***	**3**	**9.78**	**0.021**
*Varanus acanthurus*	NA		
*Varanus glebopalma*	NA		

*Note:* GLMMs were only produced for species with known nocturnal activity. Species for which tests were significant are highlighted in bold.
